# QuADRANT: a study on uptake and implementation of clinical audit of medical radiological procedures in Europe—expert recommendations for improvement, endorsed by the ESR

**DOI:** 10.1186/s13244-023-01416-7

**Published:** 2023-05-12

**Authors:** David C. Howlett, Adrian P. Brady, Monika Hierath, Jonathan Clark, Wolfgang Wadsak, Francesco Giammarile, Núria Jornet, Mary Coffey

**Affiliations:** 1 Eastbourne Hospital, King’s Drive, Eastbourne, BN21 2UD East Sussex UK; 2 Mercy University Hospital, University College Cork, Cork, Ireland; 3 European Society of Radiology, Vienna, Austria; 4 EANM - European Association of Nuclear Medicine, Vienna, Austria; 5 Department of Biomedical Imaging and Image-Guided Therapy, Medical University of Vienna, Vienna, Austria; 6 European Association of Nuclear Medicine, Centre Leon Berard, Lyon, France; 7 Servei de Radiofisica I Radioprotecció, Hospital de La Santa Creu I Sant Pau, Barcelona, Spain; 8 Discipline of Radiation Therapy, School of Medicine, Trinity Centre for Health Sciences, St. James’ Hospital, Dublin, Ireland

**Keywords:** Clinical audit, Patient centred care, Diagnostic radiology, Radiotherapy, Nuclear medicine

## Abstract

**Background:**

QuADRANT was a study funded by the European Commission to evaluate clinical audit uptake and implementation across Europe, with an emphasis on clinical audit as mandated within the BSSD (Basic Safety Standards Directive).

**AIMS:**

QuADRANT objectives—obtain an overview of European clinical audit activity; identify good practices and resources, barriers and challenges; provide guidance and recommendations going forwards; identify the potential for European Union action on quality and safety in the three core project specialties, radiology, radiotherapy and nuclear medicine.

**Findings and recommendations:**

QuADRANT identified that developments in national clinical audit infrastructure are required. National professional societies can be pivotal in improving clinical audit implementation, but resource allocation and national prioritisation of clinical audit are needed in many countries. Lack of staff time and expertise are also barriers. Enablers to enhance clinical audit participation are not widely employed. Development of hospital accreditation programmes can facilitate clinical audit uptake. An active and formalised role for patients in clinical audit practice and policy development is recommended. There is persisting variation in European awareness of BSSD clinical audit requirements. Work is needed to improve dissemination of information on the legislative requirements relating to clinical audit in the BSSD and in relation to inspection processes to ensure these include clinical audit and that they encompass all clinics and specialties involved in medical applications using ionising radiation.

**Conclusion:**

QuADRANT provides an important step towards enhancing clinical audit uptake and implementation across Europe and improving patient safety and outcomes.

## Key points

- QuADRANT was a multiprofessional study, on behalf of the European Commission, examining clinical audit across three core specialties—radiology, radiotherapy and nuclear medicine—with an emphasis on radiation protection.
- QuADRANT aimed to provide an overview of the status of European clinical audit uptake and implementation and to identify barriers and good practices.
- QuADRANT recommendations include increased prioritisation and resourcing of clinical audit, use of enablers, motivation of staff and developing the role of the national professional societies in clinical audit uptake and promotion.

## Patient summary

Clinical audit involves systematic review of clinical practices against agreed standards, with modification of practices as required and application of new standards when necessary. Clinical audit is a core component of clinical governance and has an important role in improving patient care.

The European Council Basic Safety Standards Directive (BSSD), 2013/59/Euratom lays down standards for radiation protection. Importantly, the BSSD mandates clinical audit activity “according to national procedures” and therefore this is a key area for all facilities utilising medical ionising procedures, with particular reference to European radiology, radiotherapy and nuclear medicine departments.

This paper describes the QuADRANT project, undertaken on behalf of the European Commission and led by the European Society of Radiology (ESR) working with partner societies, the European Society of Radiotherapy and Oncology (ESTRO) and the European Association of Nuclear Medicine (EANM). QuADRANT aims: provide an overview of clinical audit activity across Europe; identify good practices and resources, barriers and challenges to clinical audit uptake and implementation; identify the potential for further European Union action on quality and safety in the three core project specialties. The project successfully concluded and provided a set of recommendations for the European Commission in Summer 2022.

In many countries developments in national clinical audit infrastructure are necessary, requiring increased resourcing and prioritisation of clinical audit at all levels in the health care system. Development of hospital accreditation and health care professional certification and education programmes are recommended. Importantly QuADRANT recognises the key role patients can play in clinical audit practice and process development and an active and formalised role for patients in this area is recommended.

## Introduction

Clinical audit is recognised as a core component of clinical governance in modern health care and is an important quality improvement tool, enhancing patient care. Effective and integrated processes of clinical audit can also be of benefit to staff within departments, reinforcing confidence in clinical practices and improving the safety and quality culture within the department. In recognition of its importance in patient care and safety, clinical audit practice in support of radiation protection was mandated within the European Council BSSD, 2013/59/Euratom [1], and had to be transposed into Member State legislation by February 2018.

The BSSD mandates the use of clinical audit *“in accordance with national procedures”*, thereby providing leeway for implementation according to the resources available within individual Member States [1]. It is recognised that clinical audit as mandated within the BSSD should form part of a wider clinical audit infrastructure within national health care systems.

Clinical audit is distinct from regulatory audit and inspection. Regulatory audit verifies that practice is compliant with BSSD regulations and ensures that clinical practice correctly reflects the policies and procedures of the employer. Regulatory audit is not a BSSD requirement and does not replace inspection, but it does allow the employer to check compliance with regulatory requirements. Inspection is an investigation carried out by, or for, a national competent authority to verify compliance with national legal requirements, including the need for clinical audit. Clinical audit does not replace the need for inspection [2, 3].

Diagnostic and therapeutic quality and safety challenges have been identified in relation to ionising radiation exposure within three medical specialties in particular, namely diagnostic (including interventional) radiology, radiotherapy and nuclear medicine. Previous work by the European Commission in 2007/2008 revealed variable and often lacking, or minimal, practice of clinical audit within the Member States [4]. In response the European Commission published a keynote guidance document in 2009 to support Member States in improving clinical audit implementation: Radiation Protection 159, *“European Commission Guidelines on Clinical Audit for Medical Radiological Practices (Diagnostic Radiology, Nuclear Medicine and Radiotherapy)”* [5].

However, more recent work by the European Commission [6] and professional bodies, including the ESR, [7–10] has demonstrated a persisting lack of awareness of the BSSD requirements in relation to clinical audit and also continued variation in clinical audit uptake and implementation.

It is against this background, recognising the important patient and staff safety implications, that the European Commission initiated the QuADRANT project. This paper provides an overview of the project structure and objectives and presents the QUADRANT findings and recommendations for the use of the European Commission going forwards.

## QuADRANT—project overview, structure and aims

The original European Commission tender specification (No ENER/D3/2019 – 231 – 2) published in 2019, was entitled *“Constant Improvement in Quality and Safety of Radiology, Radiotherapy and Nuclear Medicine Through Clinical Audit”*.

The project had specific objectives:

(i) Review the status of implementation of clinical audit within the Member States.
(ii) Identify good practices in the Member States and available guidance and resources for clinical audits at national, European and international level**.**
(iii) Provide further guidance and recommendations on improving the implementation and integration of clinical audits into national health care systems**.**
(iv) Identify potential for further coordinated European Union action on quality and safety of radiology, radiotherapy and nuclear medicine.

The ESR was successful in the tender application process, leading a consortium of experts working with partner societies, ESTRO and EANM. The acronym QuADRANT was chosen for the project—**Q**uality Assurance Through Clinical **A**udit in **D**iagnostic (including Interventional) Radiology, **R**adiotherapy and **N**uclear Medicine (including **T**herapies).

The focus of the project was European clinical audit uptake and implementation, with an emphasis on clinical audit as mandated within the BSSD. Involvement of three European professional societies involved in high dose medical radiation procedures was considered important. The QuADRANT project would also link to other European Commission projects relating to ionising medical procedures and patient safety, in particular the SAMIRA initiative [11].

The QuADRANT project team and consortium were supported by a Steering Group and Advisory Board with multiprofessional and key stakeholder representation. Professional bodies and societies were represented alongside key European and international organisations, including the HERCA, IAEA and the WHO.

QuADRANT was launched in January 2020, with a proposed duration of 30 months and involved the European Union (EU) 27 Member States and four additional countries (Norway, Iceland, Switzerland, and the UK). The Covid-19 pandemic did impose restrictions on some QuADRANT components, in particular requiring online rather than face-to-face interactions at meetings/workshops but was completed on schedule.

QuADRANT comprised two workshops, a pan-European survey, expert interviews and a literature review leading to a final guidance/recommendations document for the use of the European Commission:

(i) The first workshop, held as a series of online webinars in December 2020, involved key individuals involved in clinical audit and radiation protection at national, European and international level. The workshop (> 100 invited attendees) started with a detailed explanation of the aims and methodology of the QuADRANT project and then provided an overview of clinical audit in Europe presenting existing examples of good practice in clinical audit.
(ii) The core, central, part of the project involved a Main Survey distributed to representatives of the EU 27 + 4 health authorities, national auditing organisations, radiation protection competent authorities and the three professional societies. The survey aimed to identify good practices, available guidance, resources, barriers and challenges to clinical audit uptake and implementation across Europe. There were 83 responses to the survey with at least one response from each of the EU 27 + 4. The survey was supplemented by a series of expert interviews (experts in clinical audit and/or radiation protection) and a comprehensive supporting literature review.
(iii) The second workshop was held online as two webinars in January 2022 (> 100 invited attendees), during which the survey results were reviewed and discussions centred around improving clinical audit uptake and implementation across Europe and optimum approaches to the process of clinical audit including the structure and expertise of the audit team.
(iv) The final guidance and recommendations document summarising the project was submitted for the consideration of the European Commission and was accepted by the Commission in July 2022. This document has been published in 2023 as part of the European Commission Radiation Protection series [12].

## QuADRANT—findings and recommendations

The final QuADRANT guidance and recommendations document presented its findings and conclusions in eight sections.

### Clinical audit practice and prioritisation at national level

Establishing a national body with responsibility for clinical audit is an important step in the development and oversight of a successful national clinical audit programme and creation of a national body has occurred in many countries across Europe (*n* = 23). A common national reference document with clear guidance can help countries organise and develop clinical audit infrastructure. A national body can also utilise governmental/health authority links and thereby potentially access funding for clinical audit and influence health care policy.

The national body can work with other partners, in particular the national professional societies, to develop and share good practice, guidance and policies in clinical audit. Clinical audit guides do exist in the three core project specialties [13–15] but are not widespread within the EU27 + 4. In 17 countries a clinical audit guide/manual has been developed in at least one of the three specialties, a guide is in development in four countries, 11 countries stated no manual/guide to be in place.

Another important role for a national body is the development of effective systems of data collection, particularly at national level; these allow benchmarking of data and the development of key quality indicators. It is clear from the survey and workshops that some form of data collection exists in most countries, but this is variable across specialties and countries and the collection of both quantitative and qualitative data occurs in only a minority of countries (*n* = 5). Digitised, “real time”, data collection is rare. Benchmarking of data and the development of quality indicators was identified in a majority of countries to some extent but was again variable within health care systems and across specialties.

As well as working with national professional societies a national clinical audit body is well placed to develop relationships with other relevant European and international organisations. In only a minority of the EU 27 + 4 is there formalised involvement with other external organisations, with no involvement at all reported in eight countries. Levels of communication and collaboration with other professional societies at national level are also variable.

It is important to acknowledge the significant impact of Covid-19 on health care resources and funding and this has impacted on clinical audit practice amongst many other areas. A lack of funding was identified within QuADRANT as a major obstacle in developing clinical audit at all health care levels. Although specific clinical audit funding was described via several mechanisms (one-off government/hospital/national professional society funded projects, government supported development of auditing body/society, salaries) these occur in only a minority of countries and are often unstructured and ad hoc in nature.

It is thought likely that for the majority of countries in Europe, resource allocation and dedicated funding for the further development of clinical audit infrastructure will need to be identified.

### Regulatory control—clinical audit and the BSSD

The BSSD provides the legal framework mandating participation in clinical audit in all facilities involved in medical ionising exposure, considering local and national requirements and procedures. QuADRANT identified a continued lack of awareness within national and local health care systems of the need to undertake clinical audit as mandated within the BSSD, highlighting the need for ongoing promotion, and raising of awareness amongst health care professionals and administrators of the BSSD and its clinical audit requirements.

Most European countries have an established process of hospital inspection by the relevant national radiation competent authority, although in a significant proportion of these countries (*n* = 10) the inspection process does not involve assessment of BSSD-related clinical audit. How regulatory inspection and clinical audit interact is an area needing to be addressed by the appropriate authorities.

Clinical audit should be undertaken within departments that utilise ionising radiation outside of radiology/radiotherapy/nuclear medicine (for instance cardiac catheterisation laboratories, urology, orthopaedic theatres), and within private radiology/radiotherapy/nuclear medicine facilities. The main QuADRANT survey identified that in only around 50% of countries is participation in clinical audit mandatory in these two areas of health care provision. Again, this is an area which will need attention by the appropriate authority.

### Development of infrastructure—the role of the national professional societies

The QuADRANT main survey, workshops and expert interviews all highlighted the key and potentially pivotal roles of the national professional societies in the successful development and implementation of a national clinical audit programme. Currently, national professional societies in many countries (*n* = 22) are involved in the development of good practice guidance in clinical audit, although this involvement varies by country and specialty. There is national professional societal involvement in external direction of departmental internal audit (the national society coordinates audit across multiple departments, *n* = 13) or an external audit programme (a team of external auditors visit hospitals/departments, *n* = 9) but only in a minority of countries. Other models of external audit are common with the process led by another national auditing organisation (*n* = 15), an auditing company working under licence (*n* = 8) or by another European/international organisation (*n* = 5).

The development of clinical audit infrastructure at national professional societal levels is complex and resource intensive and includes administrative support, development of functional information technology systems for communication with membership and data collection, training professionals (auditors) and engagement with health care administrators.

Lack of resources was identified within QuADRANT as a major barrier to effective national professional societal involvement in clinical audit. Resources would include not only funding but also development of staff expertise; prioritisation of resources in these areas was considered important to allow necessary developments in societal clinical audit infrastructure. These developments could in turn facilitate:

- Improved collaborative working with other national, European and international organisations and development of external national audit processes.
- Development of national quality indicators, audit manuals and guidance.
- Training of auditors (who should ideally be active professionals in the audited specialty) and provision of national expertise and leadership in clinical audit.
- Development of educational materials and teaching on clinical audit at health care professional undergraduate and postgraduate levels.

### Barriers and enablers

Several perceived barriers to clinical audit uptake and implementation were identified within the QuADRANT Main Survey and further reflected within the expert interviews and workshops.

These included:

- Insufficient funding at all levels within the health care system
- Low national and hospital priority
- Lack of time (for auditors)
- Lack of expertise amongst clinical staff

Various types of enabler can be utilised to facilitate or enhance clinical activity; these might include:

- Direct or indirect remuneration (salary for individuals, hospital/organisational financial support).
- Allowing (funded) time for clinical audit in auditor work schedules.
- Enhanced hospital accreditation or individual health care professional certification.
- Enhanced access to staff/equipment.
- Use of academic reward/recognition.

QuADRANT demonstrated that enablers were in use in only a minority of European countries. The use and/or need for enablers of clinical audit will be guided by the requirements and available resources of individual countries. The introduction of enablers can be utilised to promote and encourage individual/hospital engagement in clinical audit and their wider European implementation does merit consideration.

### Accreditation of hospitals and certification of health care professionals

Accreditation of hospital service provision can provide a marker of quality for external use and for patients. QuADRANT revealed that although there are established systems of hospital accreditation in many European countries, these are usually voluntary and where they occur evidence of participation in clinical audit is usually not required. In countries where a hospital accreditation system is in place in only 11/24 respondent countries is evidence of clinical audit participation required as part of the accreditation process.

Likewise, participation in clinical audit does form part of the certification/health care professional registration process in only a minority of countries (*n* = 9), evidence of clinical audit participation is not a requirement for continuing professional certification in the majority (*n* = 17).

Both hospital accreditation and health care professional certification schemes require resource allocation and the wider question of compulsory or voluntary involvement does need to be considered. Hospital accreditation schemes can be effective in ensuring, maintaining and benchmarking quality of service provision and if utilised should include a robust assessment of clinical audit involvement.

Regular clinical audit should be recognised as a core component of providing a high quality and safe service. It demonstrates a commitment by a centre to constant review and improvement and motivates staff to take an active role in quality assessment and improvement.

### Education of health care professionals

It became clear during QuADRANT that only a minority of countries have clinical audit teaching fully integrated into health care professional education/training programmes (*n* = 3). Barriers were identified, including a lack of understanding of the purpose, principles and benefits of clinical audit, absence of an audit culture and a lack of trained professionals/educators.

There is a need to embed clinical audit teaching within both undergraduate and postgraduate educational programmes. This can be facilitated by sharing resources, guidance and best practice between countries, national professional societies and educational institutions and cooperation between regulators, clinical staff and educators should be encouraged.

### Patient involvement

Throughout the QuADRANT project the importance of patient involvement in clinical audit was emphasised. Direct or indirect patient involvement in local/national clinical audit projects but also in the development of local/national clinical audit policy/guidance is strongly encouraged. The Main Survey and subsequent discussion within the project workshops revealed that in the majority of European countries (*n* = 21) no opportunities exist, formalised or otherwise, for patient input into clinical audit.

Access to patient health care records for data for use in clinical audit is a common requirement and in many countries such access can occur for this specific purpose without the need for formal patient consent. However, in a significant proportion of countries (*n* = 7) formal consent is a prerequisite for access to patient data and this can act as a barrier to timely data collection for clinical audit purposes. An agreed and coordinated European approach on this topic would be beneficial.

### Good practices, available guidance and resources in clinical audit

One of the key aims of QuADRANT was to identify good practices, guidance documents and resources in clinical audit at national, European and international level, with a view to sharing these on an ongoing basis as appropriate across European countries, actively cross-pollinating good ideas, concepts and working practices.

It is beyond the scope of this article to include a comprehensive list; this is available in the final European Radiation Protection series publication [12], but select examples are included below:

- Radiation Protection No 159. European Commission Guidelines on Clinical Audit for Medical Radiological Practices (Diagnostic Radiology, Radiotherapy and Nuclear Medicine) [5].
- HERCA Position Paper on Clinical Audit in Medical Radiological Practices, 2019 [2].
- Addendum to the HERCA Clinical Audit Position Paper, 2021 [3]. This paper gives an update and further information on the differences between clinical audit, regulatory audit and inspection.
- ESR Esperanto Third Edition—ESR Guide to Clinical Audit in Radiology. Vienna, 2022 [16]. This guide provides a comprehensive explanation of the principles and practice of clinical audit and is accompanied by a toolkit containing a wide range of templates relating to clinical and regulatory audit for use by European radiology departments.
- Royal College of Radiologists. Auditlive 2021 [17]. An open access resource produced by the national professional society for Radiologists in the UK and containing a wide range of templates covering clinical audit topics.

The IAEA have published clinical audit, quality and improvement tools for use in radiology, radiotherapy and nuclear medicine departments:

- Quality Assurance Audit for Diagnostic Radiology Improvement and Learning (QUAADRIL). Vienna IAEA. 2010 [13].
- Quality Assurance Team for Radiation Oncology (QUATRO). Comprehensive Audits of Radiotherapy Practices: a Tool for Quality Improvement. Vienna IAEA. 2007 [14].
- Quality Management Audits in Nuclear Medicine Practices (QUANUM), second edition. Vienna, IAEA, 2015 [15].

## Conclusion

QuADRANT aimed to provide an overview of clinical audit practice and process across Europe, identifying good practices and outlining recommendations for improving clinical audit uptake and implementation both now and in the future [12].

QuADRANT can act as an important step towards enhancing clinical audit uptake and implementation across Europe and thereby improving safety and outcomes for patients. Key areas for investment and development have been identified within existing European national health care systems that can facilitate effective clinical audit integration and provide templates for national level service improvement.

## Data Availability

Detailed data on the results reported in this article can be found in the form of tables and text in the final published version of QuADRANT which can be accessed via the European Commission website.

## Abbreviations

BSSD: Basic Safety Standards Directive
EANM: European Association of Nuclear Medicine
ESR: European Society of Radiology
ESTRO: European Society of Radiotherapy and Oncology
EU: European Union
HERCA: Heads of the European Radiological Competent Authorities
IAEA: International Atomic Energy Agency
*n*: Number of countries from which response received
QUAADRIL: Quality Assurance Audit for Diagnostic Radiology Improvement and Learning
QUANUM: Quality Management Audits in Nuclear Medicine Practices
QUATRO: Quality Assurance Team for Radiation Oncology
SAMIRA: Strategic Agenda for Medical Ionising Radiation Applications
WHO: World Health Organisation
